# 核糖核苷酸还原酶和非小细胞肺癌

**DOI:** 10.3779/j.issn.1009-3419.2012.11.02

**Published:** 2012-11-20

**Authors:** 南荣 许, 辉 李, 雅文 郑, 云 阎

**Affiliations:** 1 台北，台北医学大学附设医院胸腔外科 Division of Thoracic Surgery, Department of Surgery, Taipei Medical University Hospital, Taipei, China; 2 台北，台北医学大学拇山国际癌症中心 International Cancer Institute, Taipei Medical University Hospital, Taipei, China; 3 台北，台北医学大学医学院医学系 School of Medicine, Taipei Medical University, Taipei, China; 4 台北，台北医学大学医学科技学院癌症生物学与药物研发研究所 College of Medical Science and Technology, Graduate Institute of Cancer Biology and Drug Discovery, Taipei Medical University, Taipei, China

肺癌在全世界是癌症相关造成死亡的最常见原因，全球每年死于肺癌人数占所有死亡人数的3.1%，占所有癌症死亡人数的17.6%，排第一位^[1]^。在台湾，恶性肿瘤自1982年迄今，一直居十大死亡之首位。而其中15%-20%是因肺癌而死亡，据统计2010年死于肺癌的人数占所有癌症死亡人数的20.0%（8, 209/41, 046），位居十大癌症死亡原因第一位，且有逐年增加的趋势^[2]^。癌症发生的原因非常复杂，与先天遗传、生活饮食习惯、居住环境、职业等内外因素有关，流行病学调查显示肺癌的发生和吸烟（包括二手烟）、空气污染、职业因素（如石棉）、放射线物质（如氡气）、遗传因素、慢性疾病、性别因素、病毒感染（如人乳头状病毒）或其它危险因素有关。

和其它许多癌症类似，肺癌的发生源于癌基因的激活，或抑癌基因的失活^[3]^。癌基因指的是那些使人容易得癌症的基因。通常认为原癌基因在遇到致癌物以后会变成癌基因^[4]^。大鼠肉瘤蛋白（ratsarcoma, RAS）原癌基因的突变大约造成10%-30%的肺腺癌^[5, 6]^。表皮生长因子受体（epithelial growth factor receptor, EGFR）控制着细胞的分裂、凋零，抑制血管生成和肿瘤侵蚀^[5]^。*EGFR*基因突变和扩增在非小细胞肺癌（non-small cell lung cancer, NSCLC）中很常见，因此才有采用EGFR抑制剂的治疗基础。染色体损伤会导致基因杂合缺失，这可造成抑癌基因失活。多种基因多态性和肺癌有关，包括白细胞介素-1^[7]^、细胞色素P450^[8]^、细胞凋亡的促进因子（比如caspase-8）^[9]^和DNA修复分子（比如XRCC1）^[10]^。携带这些基因多态性的人在接触致癌物质后更容易罹患肺癌。最近的研究^[11]^发现对位基因MDM2 309G也是亚洲人得肺癌的一个风险因素。

核糖核苷酸还原酶（ribonucleotide reductase, RR）在将核苷酸双磷酸转化为2' -脱氧核苷酸双磷酸中扮演一必要的角色，接着形成脱氧核苷酸三磷酸，用来提供DNA合成和修复过程必需的RR。因此RR的表达在细胞内定位和功能中都受到高度调节^[12]^。因为RR在DNA合成中扮演如此关键的角色，所以它也是癌症治疗重要的标靶^[13]^。有关RR和肺癌的相关问题，本文综述如下。

## 1 RR的分子结构与相关研究

在RR全酶的分子中，包括一个α大次级单位和一个β小次级单位组合成一个α_2_β_2_异四分子，RR的活性需要这样的分子组成^[12]^。在人类，RR已被确认具有一个大次级单位（M1）和两个小次级单位（hRRM2和p53R2）^[14]^。大次级单位（hRRM1）包括基质和异位作用位置，可以控制RR全酶活性和基质特异性^[15, 16]^，RR次小分子形成两个对等中心含铁双核，这使得酪氨酸自由基稳定，可在催化时启动电子传递^[15, 17]^。RR两个小次级单位（hRRM2和p53R2）的蛋白质序列有80%的相似度^[14]^。试管测定发现组合成p53R2的蛋白质和hRRM2都会和hRRM1作用而形成一全酶，使得胞嘧啶核苷酸双磷酸（CDP）转化成脱氧胞嘧啶核苷酸双磷酸（dCDP）^[18, 19]^。双铁双酪氨酸关键中心被认定是RR活性中心，位于p53R2和hRRM2共同凹袋结构结合位置^[20]^，使用一合成八胜肽分子抑制RR活性，发现p53R2会经由与hRRM2一样的主控结合位置和hRRM1结合^[20]^。

p53R2和hRRM2是RR两个小次级单位，两者有一些不一样的特征，p53R2一直被认为是p53核蛋白的转录标靶^[14, 21, 22]^。而在转录时hRRM2是被细胞周期有关因子如NF-Y^[23, 24]^和E2F^[25, 26]^所调控。在正常生理情况下，hRRM1和p53R2可以在G_1_-G_0_时被探测出来；但是在不分裂的休息细胞中hRRM1似乎不会去结合p53R2或涉入脱氧核糖核苷酸三磷酸（dNTPs）的合成^[21, 26]^。研究^[27]^发现不增殖细胞并不具有RR活性，造成细胞中脱氧核糖核苷酸三磷酸（dNTPs）浓度较低；而在增殖细胞中，hRRM2在细胞位置和表达则与S期有关^[27, 28]^。

在野生型p53细胞中，面对DNA受损的反应，p53R2（而不是hRRM2）会参于DNA修复。然而，当p53没有功能，在面对UV照射时，hRRM2能够补充p53R2功能^[29]^。由于p53R2和hRRM2在结构和功能上的不同，激起许多学者对RR两个小次级单位更进一步的研究。

## 2 RR和癌化研究

研究^[30]^发现癌细胞成长、侵犯和进一步转移可能涉及成长因子Ras/Raf/mitogen被活化蛋白激酶的信号传递路径，这需要一些相关基因被活化。合成的小老鼠核糖核苷酸还原酶小次级单位R2（与人类的M2同质）过度表达时会造成膜相关Raf-1表达增加30%、MAPK-2活性增加70%以及Rac-1活性增加3倍，造成在BALB/c 3T3和NIH 3T3细胞会有很明显的转移潜力增加^[31]^。这些发现显示R2蛋白不仅是RR的一个速率限制因子，而且能够和一些致癌基因包括*v-fms*、*v-src*、*A-raf*、*v-fes*、*c-myc*和*ornithine decarboxylase*合作，提升恶性转型和癌化作用^[32]^。有趣的是，小老鼠和人类细胞株的基因转殖实验^[20, 33]^都发现核糖核苷酸还原酶大分子M1（小老鼠是R1）具有抑制恶性潜力的作用。最近Rahman等^[34]^发现以小白鼠为实验模型，将RRM2以RNA干扰（RNA interference, RNAi）剔除，会强烈抑制头颈部鳞状细胞癌细胞株和NSCLC细胞株的生长，并且将针对RR次级单位M2（RRM2）的短干扰RNA（short interfering RNA, siRNA）纳米粒子由静脉打入，会抑制肿瘤侵犯并抑制细胞的增生，进而造成细胞凋亡。有关p53R2分子作用机制，Zhang等^[35]^针对p53R2的受酶特征和抑制p53R2的试管研究发现，p53R2和hRRM2对hRRM1的结合力不同，hRRM2对hRRM1的结合力是p53R2对hRRM1的结合力的4.76倍，而RR抑制剂Triapine，则对p53R2和hRRM2的作用强度一样。这些研究可以对癌症治疗有一新的指引方向^[18]^。Zhang等^[35]^发现p53R2抑制人类癌细胞增殖，是通过上调p21、下调cyclin D1，使细胞生长循环停止，因此，p53R2不仅在修补DNA受损，而且也在人类癌细胞增生中扮演关键角色^[35]^。Piao等^[36]^最近的研究发现MEK2可通过与p53R2作用，调节RR活性，如减少MEK2的活性，使RR活性明显减少，说明p53R2受到MEK2-dependent途径的调解具有特异性。

## 3 RR在NSCLC患者的相关转译研究

### 3.1 RRM1、RRM2和p53R2表达在临床研究中的意义

Bepler等^[37]^探讨RRM1和PTEN表达对NSCLC患者预后的临床意义，发现RRM1的表达与PTEN表达和RRM2表达明显相关，而RRM1和PTEN高表达患者存活较久且疾病复发较晚。多元变项分析发现RRM1高表达是预测长期生存的因素，与肿瘤分期、生活状态和体重减轻无关。Zheng等^[38]^研究187例仅接受手术治疗的NSCLC，发现RRM1高表达的病患（> 120个月）比RRM1低表达的病患（54.5个月）的存活率好，且具有统计学差异。由于RRM1一直被学者认为是细胞内化学药剂吉西他滨的目标，RRM1在许多类型肿瘤病患中都被研究过，例如NSCLC、胰脏癌、乳腺癌、胆道癌。当这些病患使用吉西他滨治疗时，RRM1 mRNA的表达和基因的变异都会与这些病患的临床预后有关^[39]^。而Zhou等^[40]^报道使用hRRM2单克隆抗体进行免疫组织化学染色发现，在人类结肠癌、胃癌、肝癌、肺癌、胰脏癌和乳腺癌中hRRM2均有较强染色。研究结果建议可使用此抗hRRM2单克隆抗体做RR的研究和作为肿瘤癌化标记物。Simon等^[41]^最近的研究显示，针对NSCLC末期病患，可根据ERCC1和RRM1表达高低调制不同的化疗治剂，如RRM1表达低，则使用吉西他滨；RRM1表达高，则使用长春瑞滨或多西他赛；ERCC1表达高，则使用多西他赛；ERCC1表达低，则使用卡铂。如此量身打造的化学治疗会比一般常规使用化学治疗得到明显较长的存活率。至于p53R2表达在NSCLC中的临床研究，文献并不多见。Uramoto等^[42]^研究结果不支持p53R2表达在NSCLC中可以发挥重要的预后作用，p53R2介导的DNA修复途径可能负责控制肺癌的增长。然而Hsu等^[43]^研究发现p53R2蛋白表达在早期NSCLC中与癌细胞分化有负向相关。p53R2阳性表达明显有较好的存活率（*P*=0.022）。这点和Uramoto等^[42]^报道不同。因此，我们认为p53R2表达在晚期和早期NSCLC病患中可能扮演不同角色。有趣的是在食道癌中也有同样的发现，学者报告在食道癌和口腔癌中p53R2表达与肿瘤侵犯性、淋巴腺转移和肿瘤大小有关^[44, 45]^，而p53R2在晚期食道癌比早期病患中表达较高^[44]^。Hsu等^[46]^为了探讨p53R2和hRRM2在肿瘤进行的角色，以免疫组织化学在染色组织微列阵中，探讨p53R2和hRRM2蛋白在NSCLC中的表达。对92例早期NSCLC病例进行研究，发现25例表达hRRM2，占27.2%；而42例表达p53R2，占45.6%。*Kaplan-Meier*分析显示p53R2阴性表达病患比p53R2阳性表达病患的平均整体存活天数短（660 d *vs* 900 d, *P*=0.025）。另外发现hRRM2阳性表达病患比hRRM2阴性表达病患的平均整体存活天数短（660 d *vs* 960 d, *P*=0.044）。此外，p53R2+/hRRM2-或p53R2-/hRRM2-病患比p53R2-/hRRM2+或p53R2+/hRRM2+病患的存活率高（*P*=0.003）。研究^[46]^发现p53R2蛋白表达在早期NSCLC中是一个有利预后因素和预测复发生物因子，p53R2蛋白表达在早期肺癌似乎比hRRM2更重要。p53R2蛋白表达不仅可作为整体存活率的独立生物因子，也可以当作肿瘤复发的指示剂。

### 3.2 有关RR抑制剂的研究

Tu等^[47]^1999年开始使用小白鼠做研究，以RRM1反义：GTI-2501，作为RRM1抑制剂，针对各种不同人类肿瘤细胞株进行试验，包括肺癌、乳癌、结肠癌、肾脏癌、卵巢癌、胰脏癌及皮肤癌。GTI-2501是20 mer反义寡核苷酸相对位于RRM1的编码区。试管研究发现GTI-2501会降低RRM1 mRNA和蛋白的含量。此外，GTI-2501会抑制人类肺癌、肝癌、卵巢癌、脑癌、黑色素瘤、乳癌、胰脏癌细胞的成长。生物体研究也发现GTI-2501明显抑制裸鼠的结肠癌、胰脏癌、肺癌、乳癌、肾脏癌、卵巢癌、黑色素瘤、星形胶质母细胞瘤的生长。以GTI-2501治疗人类乳腺癌和小白鼠肾肿瘤会造成肿瘤完全消退。GTI-2501可抑制裸鼠黑色素瘤细胞转移到肺部并延长裸鼠淋巴瘤的存活率。这些结果说明GTI-2501可作为选择性和特定的抗癌剂^[48]^。Shibata等^[49]^针对末期转移肿瘤，使用RRM2抑制剂GTI-2040联合奥沙利铂和卡培他滨，进行第一期临床试验，认为这是可行的治疗方法。

Klisovic等^[50]^针对成人复发/难治性急性髓细胞白血病，使用RRM2反义：GTI-2040，抑制RRM2，联合高剂量阿糖胞苷进行第一期临床试验。2001年Orr等^[51]^以GTI-2040进行第一/二期临床试验，并针对肾脏癌和大肠癌作为单一治疗药剂进行第二期临床试验。但在2008年Stadler等^[52]^针对肾脏癌病患，使用GTI-2040和卡培他滨进行第一/二期临床试验，因为RRM2在细胞中的表达出现变异的数据，认为还需进一步研究。Leighl等^[53]^针对末期NSCLC，使用GTI-2040联合多西他赛进行第一/二期临床试验，发现结果并不优于以前单独使用多西他赛治疗NSCLC。

Aye等^[54]^在试管内针对3种哺乳类细胞株研究，发现Triapine可以在30 min内直接对p53R2内铁螯合，使p53R2失去活性。Mortazavi等^[55]^以Triapine长期灌注和固定剂量吉西他滨联合应用于末期固体肿瘤，进行第一期临床试验，探讨Triapine最大耐受剂量、安全性、药代动力学、药效学和有效性，结果发现在30例病患中，有1例部分反应，15例病况稳定。而Triapine已在进行第二期临床试验^[54]^。总之，有关RR抑制剂的研究，目前学者不管在基础或临床的研究都持续在努力，期待对癌症治疗提供另一种有利的选择。

## 4 展望

RR的研究，经由基础研究对结构分子的了解，到研究探讨大次级单位（RRM1）、小次级单位（RRM2、p53R2）和各类癌症的相关性，到RR抑制剂（包括如化疗制剂：羟基脲、吉西他滨；铁螯合剂：Triapine；以及反义寡核苷酸：GTI-2501和GTI-2040），进行临床前期的第一/二期试验的系列研究，为人类提供一条对抗癌症可行的路径。RR可作为抗癌的标定物，因为它是DNA合成和修补的关键分子；在许多肿瘤和细胞株的研究中发现它常会增加表达并增加酶的活性；会和一些已知的致癌基因（*v-fms*, *v-src*, *A-raf*, *v-fes*, *c-myc*, *ODC*）一起作用促成细胞转型和癌化潜能；已知RRM2具有抗癌作用；RRM2表达增多会促使癌细胞具有侵犯潜能；RR表达增多具有抗药性，这些研究的结果让人类对抗癌症充满期待。尤其最近以纳米粒子作为RR的siRNA的运送方式正方兴未艾。而有关RR抑制剂，目前仍有一些问题需要解决，包括半衰期太短、药物抑制太快、本身有部分被抑制、会有可逆性的结合现象等，相信经由学者和临床研究的携手合作，许多问题都可以逐渐理清，为人类对抗癌症的努力做出贡献。

## References

[1] Mathers CD, Loncar D (2006). Projections of global mortality and burden of disease from 2002 to 2030. PLoS Med.

[b2] 2Department of Health, Statistical Office. http://www.doh.gov.tw/statistic/index.htm [2012-08-10]

[3] Fong KM, Sekido Y, Gazdar AF (2003). Lung cancer. Molecular biology of lung cancer: clinical implications. Thorax.

[4] Salgia R, Skarin AT (1998). Molecular abnormalities in lung cancer. J Clin Oncol.

[5] Herbst RS, Heymach JV, Lippman SM (2008). Molecular oriins of cancer: lung cancer. N Engl J Med.

[6] Aviel-Ronen S, Blackhall FH, Shepherd FA (2006). *K-ras* mutations in non-small-cell lung carcinoma: a review. Clin Lung Cancer.

[7] Engels EA, Wu X, Gu J (2007). Systematic evaluation of genetic variants in the inflammation pathway and risk of lung cancer. Cancer Res.

[8] Wenzlaff AS, Cote ML, Bock CH (2005). *CYP1A1* and *CYP1B1* polymorphisms and risk of lung cancer among never smokers: a population-based study. Carcinogenesis.

[9] Son JW, Kang HK, Chae MH (2006). Polymorphisms in the *caspase-8* gene and the risk of lung cancer. Cancer Genet Cytogenet.

[10] Yin J, Vogel U, Ma Y (2007). The DNA repair gene *XRCC1* and genetic susceptibility of lung cancer in a northeastern Chinese population. Lung Cancer.

[11] Tomoda K, Ohkoshi T, Hirota K (2009). Preparation and properties of inhalable nanocomposite particles for treatment of lung cancer. Colloids Surf B Biointerfaces.

[12] Reichard P (1988). Interactions between deoxyribonucleotide and DNA synthesis. Annu Rev Biochem.

[13] Liu X, Zhou B, Xue L (2006). Metastasis-suppressing potential of ribonucleotide reductase small subunit p53R2 in human cancer cells. Clin Cancer Res.

[14] Tanaka H, Arakawa H, Yamaguchi T (2000). A ribonucleotide reductase gene involved in a p53-dependent cell-cycle checkpoint for DNA damage. Nature.

[15] Cooperman BS, Kashlan OB (2003). A comprehensive model for the allosteric regulation of class Ia ribonucleotide reductases. Adv Enzyme Regul.

[16] Wright JA, Chan AK, Choy BK (1990). Regulation and drug resistance mechanisms of mammalian ribonucleotide reductase, and the significance to DNA synthesis. Biochem Cell Biol.

[17] Ochiai E, Mann GJ, Graslund A (1990). Tyrosyl free radical formation in the small subunit of mouse ribonucleotide reductase. J Biol Chem.

[18] Shao J, Zhou B, Zhu L (2004). *In vitro* characterization of enzymatic properties and inhibition of the p53R2 subunit of human ribonucleotide reductase. Cancer Res.

[19] Guittet O, Hakansson P, Voevodskaya N (2001). Mammalian p53R2 protein forms an active ribonucleotide reductase *in vitro* with the R1 protein, which is expressed both in resting cells in response to DNA damage and in proliferating cells. J Biol Chem.

[20] Zhou B, Tsai P, Ker R (1998). Overexpression of transfected human ribonucleotide reductase M2 subunit in human cancer cells enhances their invasive potential. Clin Exp Metastasis.

[21] Yamaguchi T, Matsuda K, Sagiya Y (2001). p53R2-dependent pathway for DNA synthesis in a p53-regulated cell cycle checkpoint. Cancer Res.

[22] Nakano K, Balint E, Ashcroft M (2000). A ribonucleotide reductase gene is a transcriptional target of p53 and p73. Oncogene.

[23] Liu X, Zhou B, Xue L (2004). Nuclear factor Y regulation and promoter transactivation of human ribonucleotide reductase subunit M2 gene in a Gemcitabine resistant KB clone. Biochem Pharmacol.

[24] Currie RA (1998). NF-Y is associated with the histone acetyltransferases GCN5 and P/CAF. J Biol Chem.

[25] Chabes AL, Bjorklund S, Thelander L (2004). S Phase-specific transcription of the mouse ribonucleotide reductase R2 gene requires both a proximal repressive E2F-binding site and an upstream promoter activating region. J Biol Chem.

[26] Xue L, Zhou B, Liu X (2003). Wild-type p53 regulates human ribonucleotide reductase by protein-protein interaction with p53R2 as well as hRRM2 subunits. Cancer Res.

[27] Engstrom Y, Eriksson S, Jildevik I (1985). Cell cycle-dependent expression of mammalian ribonucleotide reductase. Differential regulation of the two subunits. J Biol Chem.

[28] Cory JG, Sato A (1983). Regulation of ribonucleotide reductase activity in mammalian cells. Mol Cell Biochem.

[29] Zhou B, Liu X, Mo X (2003). The human ribonucleotide reductase subunit hRRM2 complements p53R2 in response to UV-induced DNA repair in cells with mutant p53. Cancer Res.

[30] Huntington JT, Shields JM, Der CJ (2004). Overexpression of collagenase 1 (MMP-1) is mediated by the ERK pathway in invasive melanoma cells: role of *BRAF* mutation and fibroblast growth factor signaling. J Biol Chem.

[31] Fan H, Villegas C, Wright JA (1996). Ribonucleotide reductase R2 component is a novel malignancy determinant that cooperates with activated oncogenes to determine transformation and malignant potential. Proc Natl Acad Sci USA.

[32] Fan H, Villegas C, Huang A (1998). The mammalian ribonucleotide reductase R2 component cooperates with a variety of oncogenes in mechanisms of cellular transformation. Cancer Res.

[33] Fan H, Huang A, Villegas C (1997). The R1 component of mammalian ribonucleotide reductase has malignancy-suppressing activity as demonstrated by gene transfer experiments. Proc Natl Acad Sci USA.

[34] Rahman MA, Amin AR, Wang X (2012). Systemic delivery of siRNA nanoparticles targeting RRM2 suppresses head and neck tumor growth. J Control Release.

[35] Zhang K, Wu J, Wu X (2011). p53R2 inhibits the proliferation of human cancer cells in association with cell-cycle arrest. Mol Cancer Ther.

[36] Piao C, Youn CK, Jin M (2012). MEK2 regulates ribonucleotide reductase activity through functional interaction with ribonucleotide reductase small subunit p53R2. Cell Cycle.

[37] Bepler G, Sharma S, Cantor A (2004). RRM1 and PTEN as prognostic parameters for overall and disease-free survival in patients with non-small-cell lung cancer. J Clin Oncol.

[38] Zheng Z, Chen T, Li X (2007). DNA synthesis and repair genes *RRM1* and *ERCC1* in lung cancer. N Engl J Med.

[39] Jordheim LP, Sève P, Trédan O (2011). The ribonucleotide reductase large subunit (RRM1) as a predictive factor in patients with cancer. Lancet Oncol.

[40] Zhou B, Phan V, Liu X (2006). Production of a monoclonal antibody against the hRRM2 subunit of ribonucleotide reductase and immunohistochemistry study of human cancer tissues. Hybridoma (Larchmt).

[41] Simon GR, Schell MJ, Begum M (2012). Preliminary indication of survival benefit from ERCC1 and RRM1-tailored chemotherapy in patients with advanced nonsmall cell lung cancer: evidence from an individual patient analysis. Cancer.

[42] Uramoto H, Sugio K, Oyama T (2006). *P53R2*, p53 inducible ribonucleotide reductase gene, correlated with tumor progression of non-small cell lung cancer. Anticancer Res.

[43] Hsu NY, Wu JY, Liu X (2010). p53R2 expression as a prognostic biomarker in early stage non-small cell lung cancer. Oncol Lett.

[44] Okumura H, Natsugoe S, Yokomalura N (2006). Expression of p53R2 is related to prognosis in patients with esophageal squamous cell carcinoma. Clin Cancer Res.

[45] Yanamoto S, Kawasaki G, Yamada S (2009). Ribonucleotide reductase small subunit p53R2 promotes oral cancer invasion via the E-cadherin/beta-catenin pathway. Oral Oncol.

[46] Hsu NY, Wu JY, Liu X (2011). Expression status of ribonucleotide reductase small subunits hRRM2/p53R2 as prognostic biomarkers in stage Ⅰ and Ⅱ non-small cell lung cancer. Anticancer Res.

[47] Tu GC, Tu X (2001). GTI-2501. Lorus Therapeutics. Curr Opin Investig Drugs.

[48] Lee Y, Vassilakos A, Feng N (2006). GTI-2501, an antisense agent targeting R1, the large subunit of human ribonucleotide reductase, shows potent anti-tumor activity against a variety of tumors. Int J Oncol.

[49] Shibata SI, Doroshow JH, Frankel P (2009). Phase Ⅰ trial of GTI-2040, oxaliplatin, and capecitabine in the treatment of advanced metastatic solid tumors: a California Cancer Consortium Study. Cancer Chemother Pharmacol.

[50] Klisovic RB, Blum W, Wei X (2008). Phase Ⅰ study of GTI-2040, an antisense to ribonucleotide reductase, in combination with high-dose cytarabine in patients with acute myeloid leukemia. Clin Cancer Res.

[51] Orr RM (2001). GTI-2040. Lorus therapeutics. Curr Opin Investig Drugs.

[52] Stadler WM, Desai AA, Quinn DI (2008). A Phase Ⅰ/Ⅱ study of GTI-2040 and capecitabine in patients with renal cell carcinoma. Cancer Chemother Pharmacol.

[53] Leighl NB, Laurie SA, Chen XE (2009). A phase Ⅰ/Ⅱ study of GTI-2040 plus docetaxel as second-line treatment in advanced non-small cell lung cancer: a study of the PMH phase Ⅱ consortium. J Thorac Oncol.

[b54] 54Aye Y, Long MJ, Stubbe J. Mechanistic studies of semicarbazone triapine targeting human ribonucleotide reductase *in vitro* and in mammalian cells: Tyrosyl radical quenching not involving reactive oxygen species. J Biol Chem, 2012. [Epub ahead of print]

[b55] 55Mortazavi A, Ling Y, Martin LK, *et al*. A Phase Ⅰ study of prolonged infusion of triapine in combination with fixed dose rate gemcitabine in patients with advanced solid tumors. Invest New Drugs, 2012. [Epub ahead of print]

